# Extra Supplement—The Nursing Mirror

**Published:** 1889-07-20

**Authors:** 


*The Hospital,*
JULY 20, 1889.

[*Extra Supplement*

### The Nursing Mirror.

All Contributions for this Supplement should be addressed to the Editor, THE HOSPITAL, 139-140, Salisbury Court, Fleet Street, London, E.C., and should have the word "Nursing" plainly written in left-hand top corner of the envelope.

### En Passant.

NURSES' FRIENDS.—We really must thank Mr. Junius S. Morgan for the generosity he has ever displayed towards nurses, especially in the matter of his latest gift to the bonus fund of the National Pension Fund. From the very outset he has taken the warmest personal interest in the fund, and his encouragement and never-failing belief in the goodness of nurses, their thrift and devotion to duty, has had much to do with its wonderful success. The fact that not a single penny of the nurses' payments has been used in business or initial expenses, is one which cannot be too widely known or appreciated. With the Princess of Wales for their president, and city merchants for their friends, the members of the Pension Fund are indeed doubly blessed.

REGISTRATION.—We print on the next page the important memorial which the authorities of the great hospitals and nurse-training schools have issued on this subject. Having slain the proposals of the British Nurses' Association at a blow, it will be incumbent upon these authorities to take up the one crumb of substantiality embodied in the scheme of the promoters of the association. That is, they must secure the public, as they alone can, against the annoyances and dangers of pseudo-nurses, by making detection easy and certain. This they can easily do by determining to issue every year an Official Directory of Trained Nurses under the editorship of one of the lecturers, and the authority of a committee of representatives of the nurse-training schools. This directory should contain the name of every certificated nurse, together with her qualifications and the examinations she has passed. It should be issued free of all expense to the nurses, and ought to be published annually at a cost of not more than 1s. per copy.

MEDICAL WOMEN FOR INDIA.—The meeting at the Mansion House on July 10 was well attended, and several points regarding the aims of the Countess of Dufferin's Fund were cleared up. Sir William Hunter said that the objects of the association might be briefly stated as follows: In the first place, to create a system of medical education for women in India; in the second place, at once and continually to bring medical relief by means of female practitioners within the reach of the women of India so far as the funds of the society would permit; and, in the third place, to supply trained midwives and female nurses for women and children in hospitals and private houses. There were now 220 women in India receiving education in the medical schools of that country. In every presidency and province numerous dispensaries and several large hospitals had been founded for the relief of women and children. There were now sixty-one medical women practising in India, and of these nineteen were in direct connection with the society. Sir Arthur Lyall afterwards described the progress of the association, especially in Northern India, and Sir M. Grant Duff spoke of the work of female medical education and relief in Southern India. The Lord Mayor then announced the receipt of several donations, including 500 guineas from the Drapers' Company, 50 guineas from the Princess of Wales, and 50 guineas from the Baroness Burdett-Coutts. It will be seen from Sir William Hunter's statement that the term "medical women" is used to include nurses and midwives.

MORAL ATMOSPHERE.—If the nurse is responsible for the atmosphere of her ward, so is the matron responsible for the moral atmosphere of the nurses' home. And trifles have great force in keeping this moral atmosphere pure and invigorating. Tidy rooms, methodical habits, personal neatness, may not be numbered amongst the primary virtues, but the nurse who is trained in them has learned so much force of character. The matron who has very high aims and a grand ideal will not achieve much unless she works through the lesser towards the larger.

MEETING AT THE MANSION HOUSE.—On Wednesday a meeting was held at the Mansion House by the British Nurses' Association, over which the Princess Christian presided. We understood that some business was to be done—a charter to be brought forward—but in reality the only resolution was one of thanks to the Princess Christian. The hall was full, the black uniform of Barts. predominating. On the platform were Mr. and Mrs. Bedford Fenwick, Dr. Pavy, Mr. Brudenell Carter, Mr. Savory, and others. After the protest printed in the morning papers, and which we reproduce this week, it is not to be wondered at there was little to be said.

PRIZE-GIVING AT THE LONDON.—There was a very merry gathering at the London on Monday, when the Duke of Cambridge presented Sir Andrew Clark with his portrait, and gave the students and nurses their prizes. Each member of the medical staff was received with cheers, and Mr. Rivington's speech called forth plenty of laughter. The successful probationers were Miss Bishop, Miss Ridal, and Miss Wheeler, who carried off the three prizes: honorary certificates were given to probationers Collinson, Herrman, Huntley and Yatman. The Duke made a pretty reference to the excellent nursing at the London, and afterwards in going round the wards he made practical enquiries about the work done there. Several matrons from other hospitals were present, and a great many old friends of the London, glad of the opportunity of wandering about the dear old wards once more. Mr. Aylward, Mr. Blomfield, and Mr. Mulvany, carried off the lion's share of the students' prizes.

NURSES AT A FETE.—There was great excitement at University College Hospital on July 9, as all the nurses who could be spared had been invited by Sir Whittaker Ellis to view a river fête from his garden at Richmond. Some fifty nurses were selected for the treat, and enjoyed a delightful drive down and a tea with strawberries and cream. Then they watched the thousands of fairy lights beginning to twinkle along the river banks, and they watched the boats hung with Chinese lanterns which glided silently down the stream. Then, just as the darkness came on and the decorations became properly resplendent, a slow, steady rain began. It was no use gathering under the trees, or trying to be gay; the rain was penetrating and depressing, and one by one the illuminations went out, the bands ceased to play, and the nurses grew silent and chilly. Supper had been ordered for ten, so had the brakes, and of the two the nurses preferred to get into the brakes and get home to the hospital. Here they were received by their comrades, and all, now again dry and cheerful, enjoyed a splendid repast. (N.B. What a pity there are not a few more governing bodies of hospitals as generous as that of University College, and what a pity there are not a few more secretaries like Mr. Newton Nixon!)

### The Proposed Registration of Nurses.

### MEMORIAL OF NURSE-TRAINING SCHOOL AUTHORITIES.

We, the undersigned, beg the favour of your insertion of the following statement, which we think it desirable to make, in view of a paragraph which has been published on the subject of the registration of nurses, in which we note with surprise the statement that the main object of the British Nurses' Association "is in conformity with a great public want and a widespread professional demand."

We would wish to point out that those who represent the largest nursing interests in the metropolis and throughout the country, and who have the most to do with the training and examination of nurses, have not only declined to take part in the association, but consider that its proposed enrolment of nurses in a common register, if carried out, would (1) lower the position of the best trained nurses, (2) be detrimental to the advancement of the teaching of nursing, (3) be disadvantageous to the public, and (4) be injurious to the medical practitioner.

We hope that a final judgment upon this important matter will be postponed until the views of those who are opposed to the aims of this association have been expressed and examined. We further consider it our duty to state that if a charter be applied for on the lines stated in the prospectus of the British Nurses' Association, we shall feel it to be incumbent upon us to offer thereto all legitimate opposition in our power.

(Signed)

St. Thomas's Hospital and Nightingale Fund Training School.

D. H. Stone, treasurer of the hospital.
Harry Verney, chairman of the Nightingale School.
W. Bowman, F.R.S., member of council of Nightingale Fund, and of council of St. John's House and Sisterhood.
W. Rathbone, trustee and member of council Nightingale Fund; president Liverpool Training School and Home for Nurses.
H. Bonham Carter, secretary of the Nightingale Fund.
J. S. Bristowe, M.D., F.R.S., senior physician of St. Thomas's Hospital, and lecturer in Nightingale School.
A. L. Pringle, matron of St. Thomas's Hospital, and superintendent of Nightingale Fund Training School.
M. S. Crossland, sister in charge of the Nightingale Training School.

Guy's Hospital and Training School.

E. H. Lushington, treasurer.
E. C. Perry, M.D.; G. Newton Pitt, M.D.: assistant physicians and instructors of probationer nurses.
J. C. Steele, M.D., superintendent and instructor of nurses.

Westminster Hospital and Training School.

Westminster, chairman.
Rutherford Alcock, vice-chairman.
J. J. Troutbeck, D.D., hon. treasurer.
Mary E. Thynne, hon. secretary of committee of management of Training School.
W. H. Allchin, M.B., physician to the hospital; Thomas Bond, F.R.C.S., surgeon to the hospital: lecturers to the nursing staff.
Mary J. Pyne, matron of hospital and lady superintendent of nurses.

St. Bartholomew's Hospital and Training School.

Norman Moore, M.D., assistant physician; Harrison Cripps, F.R.C.S., assistant surgeon: instructors of probationary nurses, St. Bartholomew's Hospital.

Charing Cross Hospital and Training School.

John B. Martin, treasurer and chairman of committee.
Stanley Boyd, F.R.C.S., senior assistant surgeon and lecturer to nursing staff.
Hughina A. C. Gordon, lady superintendent.

King's College Hospital and Training School.

Henry Wace, D.D., chairman of committee of management.
Richard Twining, treasurer.
Nathaniel Bromley, A.K.C., secretary.
John Curnow, M.D.; Nestor Tirard, M.D.: physicians to the hospital, and examiners and lecturers to the nursing staff.
Katherine H. Monk, matron.
Clara S. A. Peddie, house sister and teacher to the nursing staff.

London Hospital and Training School.

F. C. Carr-Gomm, chairman of house committee.
J. H. Buxton, treasurer.
A. Ernest Sansom, M.D.; Frederick Treves, F.R.C.S.; James Anderson, M.D.: examiners and lecturers to the nursing staff.
Eva C. E. Lückes, matron.

St. Mary's Hospital and Training School.

T. Pycroft, chairman of house and finance committee.
W. Handfield Jones, M.D., assistant obstetric physician; A. J. Pepper, F.R.C.S., assistant surgeon; S. Phillips, M.D., assistant physician; A. Q. Silcock, F.R.C.S, assistant surgeon: lecturers to the nursing staff and examiners.
E. M. Medill, matron.

St. Marylebone Infirmary and Training School.

John R. Lunn, F.R.C.S., medical superintendent.
Elizabeth Vincent, matron.

Many other metropolitan and provincial hospital authorities have already signed this memorial, and names are being sent in daily to Dr. Steele, Guy's Hospital, S.E. It is proposed to publish a complete list later on, but meanwhile considerations of space must confine it to the managers and teachers of the great London hospitals and nursing schools.

### The British Nurses' Association.

We have received an interesting account of a meeting of the council of this association, which was held at the Medical Society's Rooms, Chandos-street, W., on Friday, 12th inst., when the Princess Christian presided. The charter was submitted to the meeting, and members present were invited to express their opinions and views thereon. Several members were not slow to accept the invitation, and the character of their criticisms and their force may be realised from the fact that not only was the charter withdrawn, but it was determined to postpone an application to the Privy Council. It would therefore appear that the first echo of criticism has frightened all life out of the charter of which nurses have heard so much, and it must now be consigned to the limbo of immature schemes hurriedly prepared without adequate reason or practical knowledge of the facts to be handled. It was next proposed and ultimately decided that the association should start a general register at once, upon which should be placed the names of every woman who has subscribed half-a-crown to the British Nurses' Association—*i.e.*, every member of that body. Miss Stuart and others strongly protested against this being done, on the ground that members had been hurriedly admitted, without adequate enquiry as to their qualifications or right to the title of "nurse." Some speakers had, in the course of the meeting, complained of the admission of members without their knowledge, and generally as to the unsatisfactory character of the arrangements in these respects. Miss Stuart based her objections on this evidence, and pointed out that for the reasons stated, every self-respecting nurse must shun such a register, because to be placed upon it would injure her professional position, if it did not militate against her earning a living. Despite Miss Stuart's objections, this extraordinary proposal was adopted. Another suggestion was to form a committee of "men of business" to manage the affairs of the association, as the absence of business qualities was found to work awkwardly under the present arrangements. It appeared that when the solicitor was not present (and he could not always attend the meetings), business was apt to fall into arrears. Surprise having been expressed at these statements, a gentleman on the platform rose to explain why the proposal was made. He pointed out that at a recent meeting there had been some money available for investment, but no one present knew how or where to place it to the best advantage. After further discussion the proposal was negatived. H.R.H. Princess Christian exercised much tact and judgment in the conduct of the proceedings, and great credit is due to her that they passed off without serious disagreement or a general shindy.

### Physiology for Nurses.

### No. II.—The Skin.

The true skin also contains the bloodvessels and nerves, and they make their presence painfully known to you every time you cut your finger deeply, for if there were no nerves you could cut it as deeply as you liked and not feel any pain; but, also, were there no nerves in your skin you would not have the sense of touch. People who get paralysed are not able to feel at all in the paralysed part, because the nerves are not in working order. Moreover, the nerves also make us blush—the sense of pleasure, or pain, or shame which begins in the brain is carried along the nerves which cause the bloodvessels to expand and contain more blood and thus because the skin of the face is thinner and clearer than it is in many parts of the body this redness is seen distinctly, and we get what we call "blushing." Darwin says that this power of blushing belongs entirely to intelligent human beings, and that neither very young children nor idiots nor any known animal has this same power—therefore, though many sensitive people who blush very easily find it inconvenient and annoying, they may console themselves with the thought that it is one of the penalties we must pay for being such superior beings. In the deepest layers of this true skin are a number of fat-cells massed together, and it is this fat which gives the smoothness and softness to the skin; and because as age advances this fat disappears and is not replaced, the skin becomes more and more wrinkled and furrowed, except in cases where people are also stout as well as advanced in years, and then this fat remains. In cases where people have been starved to death, there is no fat. The use of this fat is to give a soft, elastic cushion which prevents the parts below it from being injured; in the case of the feet, which have to bear the weight of the whole body, it makes this weight to be borne with ease and comfort, and in the palms of the hands it prevents any pain from grasping hard things. This fat is found in the largest quantities where it is most wanted and is therefore very unevenly distributed—the backs of the hands, tops of the feet, and the scalp, amongst other parts, have very little or none. Again, the skin itself is very much thicker in some parts than in others, for instance, on the palms of the hands and soles of the feet, but that is entirely due to the pressure they have to bear. If you had a serious illness which obliged you to stay in bed for many weeks, you would find your feet were very tender when you first began to try to stand on them, because the skin on the soles had not been accustomed to bearing your weight for so long, though the skin will gradually harden and thicken again. The surface of the skin has very minute openings called "pores"; these are the third kind of openings, the other two being the sweat glands and the oil glands, and through these pores it is possible to absorb fluids. You may have heard of sailors who have been shipwrecked and had to escape in boats, being tossed about in mid-ocean for days or weeks. All their stock of fresh water has gone, and they are in great risk of dying of thirst. They are surrounded by water on all sides, but they know it means terrible after-suffering and death if they drink the sea-water. However, they find that they can relieve their terrible thirst by soaking their clothes in the sea-water, and this wonderful power of absorption which their skin has enables it to take up the water, and leave the salt and other injurious matter behind, and thus their suffering is a little lessened.

The uses of the skin are seven—viz.: (1) It protects the external surface of the body and supports the internal organs; (2) by the immense number of nerves which it possesses we are able to recognise the feeling of pressure, touch, temperature, and pain; (3) it has a great deal to do with the warmth and cold of the whole body; (4) it forms sweat and oily matter out of the materials of the blood; (5) it helps the lungs in respiration; (6) it can absorb certain substances; (7) it helps to keep the blood healthy by ridding the body of impurities. There can be no question as to the protection given by the outer skin, nor as to the great amount of that protection; but with regard to the internal organs the protection is less, and, moreover, it is less important, because the internal organs do not depend on the skin for their protection. Supposing you were to remove the skin from a human being, the body would still remain whole and keep its natural shape, just as a rabbit's does when it has been skinned. The skin has the power of becoming thick and tough when it is not kept from the air and light by clothing. Our hands and faces have thicker skin than the rest of our bodies because of this exposure, and with those races who go about with very little clothing the skin becomes tough and less sensitive to cold, and thus more of a real protection to the body.

*(To be continued.)*

### The National Pension Fund for Nurses.

We are gratified at being able to state that the House Committee of the London Hospital determined to affiliate with this fund at their meeting on Tuesday last. The committee accepted in its entirety the scheme prepared, we believe, by the secretary, Mr. G. Q. Roberts, and agreed to pay one half the premium of every member of their staff who joins the National Pension Fund for Nurses. These payments will be made out of the earnings of the private nursing home attached to the hospital. The draft scheme is so excellent that we hope it may be supplied to the authorities of any hospital who may apply to Mr. Roberts, at the London Hospital, for it.

We are glad to know that hospital authorities are generally recognising that a nurse who has served the hospital for years is entitled to help to a greater extent than the payment of half the premium necessary to secure her pension. It is becoming the practice to take the premiums of a nurse aged twenty-five next birthday as a basis of calculation for a minimum pension of £15 at fifty-five years of age, under table B—half the premium (£4 18s.) being £2. 9s. in her case. A nurse joining at the same age as No. 1, but having served for ten years, would be thirty-five next birthday, and the premium for the same benefits, under the same table, is, in her case, £8 3s. Now, instead of paying half the premium of No. 2 nurse, the hospital authorities agree to pay £6 4s. on account of her ten years' service, leaving £2 9s. only to be paid by herself—*i.e.*, the same sum as that paid by No. 1—and so on in proportion for the whole staff. This plan is not only just but worthy of wide imitation and acceptance, and will, we feel sure, if it becomes general, overcome every nurse's difficulty in joining the fund.

### Everybody's Opinion.

[*Correspondence on all subjects is invited, but we cannot in any way be responsible for the opinions expressed by our correspondents. No communications can be entertained if the name and address of the correspondent is not given, or unless one side of the paper only be written on.*]

### THE LATE ACCIDENT AT THE ROTUNDA.

AN OLD PUPIL writes: In your column called "Scraps and Gleanings" for July 6th, a short notice appeared referring to the late accident at the Rotunda Hospital. It ran thus: "A patient at the Dublin Rotunda lately leaped from the window and killed herself. It is alleged that the nurse in charge was asleep." Now, I think in justice to the members of the hospital, the idea of neglect, as implied in the above cursory statement of the affair, should be withdrawn by a clear statement of the circumstances attending the occurrence. The woman was admitted to the hospital on the 30th March, being near her confinement. Pending that event, she was placed in the auxiliary or gynæcological department, as is the custom with patients arriving from a distance. Up to the 29th May, when she jumped out of the window, the patient's conduct had not in any way aroused the least suspicion in the minds of any of the officials. She was, as far as could be ascertained, considering her condition, in good health up to the time of her death. Now, in the Gynæcological Hospital it is not usual, unless under exceptional circumstances, for patients to have a special nurse. The woman was therefore, with seven other patients, in charge of a ward-nurse; the latter sleeps in the ward with the patients. With regard to the nurses sleeping in the wards with the patients, it is certainly to be regretted that the want of accommodation at the hospital necessitates such a proceeding both in the midwifery and gynæcological departments. A midwife in training sometimes occupies a separate bed in a private ward with a patient, or, as necessity requires, a bed in the public ward, where most convenient. The staff nurses in both hospitals (with the exception of the head midwife) invariably sleep in the public wards, a system which must eventually prove injurious to the strongest constitution, even allowing for the admirable system of ventilation at the Rotunda. Surely the safety of the patients and the health of the nurses would be better ensured by better and separate sleeping room being provided for the latter, who cannot possibly be expected to be on the alert asleep as well as awake, and in the interests of the patients, nurses appointed for night duty (independent of special cases) in the different wards with means of communicating with headquarters, if necessary. It is said that on the morning of the accident, about 5 a.m., the patient quietly got up, and the first intimation the nurse had of the occurrence was the noise occasioned by the fall. Certainly the nurse was asleep when the accident took place, but surely, under the circumstances, she deserves no blame. The nurse always gives assistance to any requiring it when called upon to do so during the night, otherwise she is entitled to rest, and, indeed, a night's rest without interruption is a boon not often enjoyed. It is well known that cunning is one of the chief characteristics of insanity, and the patient, in order to elude suspicion or vigilance, must have carefully watched her opportunity for escaping, as none in the ward were aware of her absence until nurse and all were awakened by the noise outside. It is much to be regretted, some say, that the windows were not secure. True, but as such accidents have been of such very rare occurrence, such precaution has not hitherto been deemed necessary. It is probable, however, that this incident is likely to lead to more secure means of protection in that direction; for, as it is absolutely necessary (in this weather particularly) to obtain all the ventilation possible where such a number of people are sleeping, the windows were of course open, and the patient had no delay in getting out. Though, compared with most modern hospitals, the internal arrangements at the Rotunda for the nurses leaves much to be desired, the teaching and training given by the medical staff in midwifery and gynæcology is unsurpassed. No pains are spared by the masters to qualify their pupils, and it is to be regretted that the accommodation at the hospital admits of but a very limited number of intern pupils. It is hoped that for the comfort and health of the nurses, the plans which have been some time spoken of for providing suitable sleeping accommodation, bathrooms and dining-hall, will soon be put into execution, and supersede the limited appointments at present at their disposal. From experience, I must say that a term at the Rotunda Hospital would amply repay anyone desirous of obtaining a thorough knowledge of the subjects taught there. Though a nurse of many years' standing, and considered successful generally in my profession, I acknowledge on facing the Rotunda course I stood amazed, at what?—my ignorance. To thoroughly understand the duties, difficulties, and dangers connected with the science of obstetrics, I think a course at some such hospital is absolutely essential, as part of the training of all nurses engaged in the nursing of women. In conclusion, I must say that I think any pupil, male or female, holding a certificate from the grand old school of Irish obstetrics may feel justly proud of the possession.

### A PLEA FOR NURSES.

A. S. writes: With regard to Greta's remarks about nurses going to Holy Communion, I can only speak for my own. They can go any day, Sunday or weekday, whenever they ask. To church they go in turn, and are never kept away by anyone but the house surgeon, who often fixes Sunday morning for extra dressings, and, of course, no matter how put out a nurse may be she must not show it, though if a pin is not handed at exactly the right angle her head is likely to come off.

### Appointments.

[It is requested that successful candidates will send a copy of their applications and testimonials, with date of election, to THE EDITOR, The Lodge, Porchester-square, W.]

CHELSEA HOSPITAL.—Miss Norbury has secured the matronship of this delightful old building. Miss Norbury trained at the Princess Alice Memorial Hospital, and then went to the Middlesex, where she acted as Sister Broderip for a year. She is the daughter of Colonel Norbury, and it is her connection with the army which has secured her her present post. Miss Turner has been appointed Sister Broderip in her stead.

CHESTER DEACONESS INSTITUTION.—Miss Vickers has been appointed superintendent of the nurses of this institution, who work so hard amongst the sick poor. Miss Vickers is well known in Chester, as she trained for three years at the infirmary there.

NEWPORT INFIRMARY, MON.—Miss Flood has just been appointed matron to this infirmary. She was trained at Guy's Hospital, where she obtained her certificate in September, 1887, since which date to the present time she has filled the appointment of sister in the female wards of the Glamorgan and Monmouthshire Infirmary, Cardiff. Miss Flood's testimonials are excellent, and we congratulate her on her appointment.

### Vacancies.

The following vacancies are announced:

**Matrons.**

Nottingham Children's Hospital, £60.
Perth Royal Asylum.

**Nurses.**

Aberdeen City Hospital (fever), £26.
Bath Trained Nurses' Home (four), £25.
Brighton Fever Hospital (head), £28.
British Home for Incurables (head.)
Chelsea Infirmary, £18.
Croydon Hospital.
Darlington Fever Hospital (night), £20.
Hampshire Nurses' Institute.
Hanwell Ophthalmic School (five), £26.
Hollond Institution, Nice.
Miller Hospital, Greenwich, £22.
National Hospital, Queen's-square, £22.
Newlands, Ryde.
Nurses' Institute, Jersey, £25.
Queen Victoria Nursing Institution, Wolverhampton (several.)
Royal Hospital, City-road, £25.
Southport Nurses' Home.
Stratford-on-Avon Hospital, £20.
St. William's Hospital, Rochester, £25.
Torquay Sanatorium, £40.
Victoria Home for Nurses, Bournemouth, £25.
West London Hospital (assistant), £24.

**Midwives.**

St. George's Dispensary, Hanover-square.
Mother's Lying-In Home, Shadwell.

**District Nurses.**

Bath Trained Nurses' Home.
1, Regent-street, Nottingham.

**Probationers.**

Bristol District Nurses' Society.
Bradford Infirmary.
Grimsby Hospital.
West Cornwall Infirmary.
West London Hospital, Hammersmith.

**Sister.**

Glamorganshire and Monmouthshire Infirmary.

### Notes and Queries.

TO CORRESPONDENTS.—1. Questions may be written on post-cards. 2. Advertisements in disguise are inadmissible. 3. In answering a query please quote the number. 4. If a private answer is desired, a stamped addressed envelope must be forwarded.

### Queries.

(44) *Male Nurses in the States.*—Could you give me the address of any institution in America which employs male nurses? *T. W. S.*

(45) *Buenos Ayres.*—Any information regarding nursing in Buenos Ayres will be welcomed by "*Vagrant.*"

(46) *Holiday Home.*—Where can a nurse spend her holidays in August at a seaside home? *S. O.* (See our advertisement pages. *Editor*).

(47) *E. A. W.* asks if there is any place where massage is taught free?

### Answers.

(43) *Chatelaines.*—Write to Messrs. Ferris, Union-street, Bristol. I got a nice one there for about five shillings. *Nurse Nora.*

(44) *Male Nurses in the States.*—Apply to the secretary, State Charities Aid Association, 21, University-place, New York.

*Nurse P.*—To secure a post at the Alfred Hospital, Melbourne, apply to the secretary.

*Miss G.*—You can be received for a few months at the Seamen's Hospital, Greenwich, and so secure the slight knowledge of nursing you desire.

*W. S. G.*—We know of no convalescent home at Margate, but there are several at Ramsgate. Try either St. Luke's Home, or St. Barnabas Home.

